# Transient osteoporosis of the hip: Physicians the occupation of at risk

**DOI:** 10.1002/ccr3.4968

**Published:** 2021-10-15

**Authors:** Abolfazl Fattah, Mahdi Abounoori

**Affiliations:** ^1^ Department of Internal Medicine School of Medicine Semnan University of Medical Sciences Semnan Iran; ^2^ Student Research Committee School of Medicine Mazandaran University of Medical Sciences Sari Iran

**Keywords:** hip pain, physician, transient osteoporosis of the hip

## Abstract

Physicians are the occupation at risk for Transient Osteoporosis of the Hip. Therefore, sudden hip pain in them should be further evaluated with magnetic resonance imaging. Conservative treatment may help avoid further injury and speed up healing.

## Graphical abstract

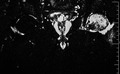

## 1 INTRODUCTION

Transient osteoporosis of the hip (TOH) is a clinical disorder with an unclear cause that generally disappears. The occupation of a physician may raise the risk of TOH in these healthcare workers. Conservative treatment may help avoid further injury, speed up healing, and reduce recovery time.

Transient osteoporosis of the hip (TOH) is a temporary clinical condition of unclear origin marked by transitory pain, impairment, and radiological abnormalities such as hip osteopenia. TOH generally resolves with conservative treatment, but it may be exacerbated by fracture or development to avascular necrosis (AVN).^1^ Although TOH is somewhat more common in males, it is most often observed in women in the later stages of pregnancy.^1^ In addition, physicians may be at an elevated risk of getting TOH due to their job.^2^ Herein, we present a TOH case physician.

## 2 CASE PRESENTATION

A 46‐year‐old male physician presented himself with sudden left hip pain, lack of weight‐bearing to the left leg, and pain radiation to the medial part of the left thigh. In his physical examination, he reported a limitation in all left hip movements, and other physical examinations were normal. The results of a complete hemogram, coagulation panel, thyroid function testing, and blood sugar monitoring were all normal. The levels of rheumatoid factor, antinuclear antibody, and C‐reactive protein were within normal limits, ruling out inflammatory arthritis‐related osteoporosis. Vitamin D levels were 35 ng/ml, and serum calcium was 9.5 mmol. The renal parameters, serum phosphorus, alkaline phosphatase, and parathyroid hormone were all within normal limits.

**FIGURE 1:**
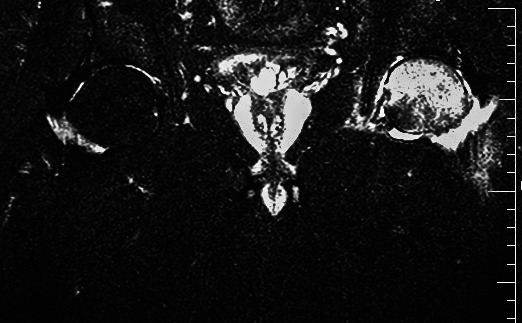
T2‐weighted MRI of the left hip: Coronal image reveals widespread bone marrow edema

Through the coronal view of the left hip's T2‐weighted magnetic resonance imaging (MRI), widespread bone marrow edema was observed, consistent with transient osteoporosis of the hip (Figure 1).

**FIGURE 2:**
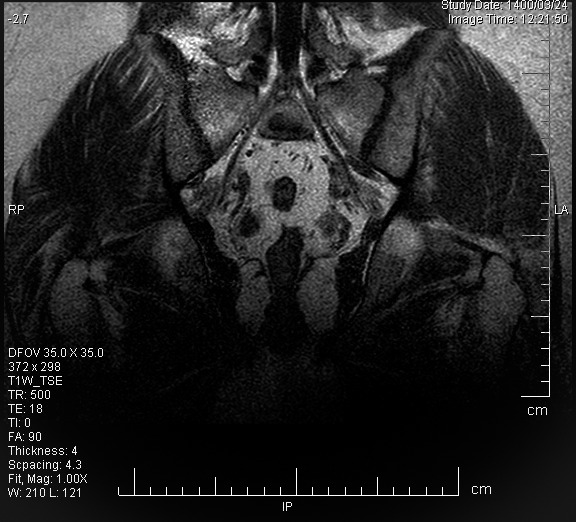
Indicating spontaneous resolution of bone marrow edema of the left hip after three‐month conservative treatment

After three months of rest and therapy with vitamin D3 supplements (50,000 IU, one pearl each week), calcium (1000 IU, one tablet each day), and Nonsteroidal Anti‐inflammatory (NSAID) medication (Naproxen 500 mg, two tablets in a day), the patient was scheduled for a follow‐up checkup. He had a good outcome from the subsequent appointment. Three months after starting a course of therapy, the latest MRI scan showed improvement in the edema of bone marrow (Figure 2).

## 3 DISCUSSION

Three phases are thought to exist in TOH. Edema caused by trauma, neurovascular dysfunction, transitory hyperemia, or microfracture causes the initial stage of acute onset hip pain. Increased bone resorption and demineralization characterize the second stage. The last stage involves the clinical and radiographic resolution of the process.^3^

It is difficult to say how common it is in an average population‐based on literature. However, one study reported that physicians accounted for 11 of the 17 (64%) individuals who participated in the TOH trial.^4^ In another study, of 15 patients with TOH, eight (53.3%) were physicians.^2^ So, it has been revealed that physicians, particularly, are associated with an increased risk of having TOH.

The etiology of TOH is unknown. It is also uncertain if TOH is a separate entity from avascular necrosis (AVN) or reflects the early stages of the disease. Trauma, infection, inflammation, degenerative process, ischemia damage, neoplasia, surgery, medications, metabolic, neurologic diseases, and pregnancy are potential causes of TOH. Other risk factors mentioned in the literature include alcohol use, steroid use, smoking, hypothyroidism, hypophosphatasia, osteogenesis imperfecta, low testosterone, low vitamin D (25‐cholecalciferol), and specific professions.^1^

The most excellent technique for demonstrating edema inside the bone is MRI, which is sensitive enough to identify TOH as early as 48 h after symptoms start^3^ and to rule out other conditions, including avascular necrosis, insufficiency fractures, infection, and malignancy.^5^ The average onset age is 40 years old, with occurrences documented between the ages of 20 and 80.^1^ The physician's profession may be regarded and studied as an independent risk factor for TOH. According to the proposed venous occlusion pathophysiology, the greater incidence of doctors may be accounted for by their lengthy durations of standing and protracted work sessions.^2^

It is critical to distinguish this disease from other hip disorders such as AVN of the femoral head, insufficiency fracture, and infection.^6^ TOH is sometimes misinterpreted as femoral head AVN. AVN has a more gradual start and the potential to develop to femoral head collapse. On the contrary, TOH has a more benign end result, as seen by spontaneous remission within 6–12 months.^6^ On MRI, T1‐weighted pictures of TOH exhibit decreased signal intensity, whereas T2‐weighted imaging indicates increasing signal intensity.^6^ The case pain was sudden and spontaneously regressed within three months. Also, MRI findings were consistent with TOH, and with the aid of MRI, other conditions such as insufficiency fracture and infection were ruled out.

Rest and analgesic medicines for pain management are used to treat TOH. In addition, TOH can be treated with bisphosphonate, teriparatide, or calcitonin treatment to speed up recovery. Core decompression surgery is an additional alternative for individuals who have not responded to medical treatment.^1^ Some studies mention that hip drilling patients usually recover in less than a month, faster than conservative therapy.^2^, ^7^

## 4 CONCLUSION

The occupation of a physician may raise the risk of TOH in these healthcare workers. Although TOH is a self‐limiting disease that usually resolves spontaneously with full recovery within several months, conservative therapy with NSAIDs, calcium, and vitamin D supplements may help avoid further damage, accelerate healing, or shorten recovery time in most instances.

## CONFLICT OF INTEREST

None declared.

## AUTHOR CONTRIBUTIONS

MA. and AF. involved in conceptualization and supervision. MA. involved in writing—original draft preparation and project administration. All authors involved in writing—review and editing. All authors have read and agreed to the published version of the manuscript.

## ETHICAL APPROVAL

Because this report involves no experiment, ethics approval is waived.

## CONSENT

One of the authors of this paper was the case, and he, with his informed consent, helped write this case report.

## Data Availability

Data sharing is not applicable to this article as no new data were created or analyzed in this study.
